# Older U.S. Adults With Nocturia Often Cannot Use the Only FDA-Approved Drugs for the Condition

**DOI:** 10.1093/geroni/igaa057.827

**Published:** 2020-12-16

**Authors:** Theodore Johnson, Kara Suvada, Laura Plantinga, Anna Mirk, Alayne Markland, Mohammed Ali, Camille Vaughan, Kathryn Burgio

**Affiliations:** 1 Emory University School of Medicine, Atlanta, Georgia, United States; 2 Emory University, Atlanta, Georgia, United States; 3 University of Alabama at Birmingham, BIRMINGHAM, Alabama, United States; 4 University of Alabama at Birmingham, Birmingham, Alabama, United States

## Abstract

Nocturia, waking from sleep at night to void, is a common, bothersome symptom for which many older adults seek treatment. In 2017, the US FDA approved the first of two desmopressin analogues indicated for nocturia. While efficacious, these drugs can potentially cause severe hyponatremia that can be fatal, particularly if older adults have comorbid conditions (e.g., congestive heart failure, uncontrolled hypertension), laboratory abnormalities (e.g., low sodium, reduced renal function) and concomitant medications (e.g., diuretics, analgesics). Using secondary data from the National Health and Nutrition Examination Survey (NHANES) to identify a sample of U.S. adults 50 years and older with 2 or more nightly nocturia episodes, we determined the prevalence of contraindications, warnings, and need for more frequent monitoring indicated by the product label. Among the sample of 1,521 older respondents, 70.0% of those with nocturia had contraindications or a need for frequent sodium monitoring. Contraindications to desmopressin analogues were more prevalent with older age (27.6%, 34.5%, and 38.8%, for ages 50-64, 65-79, and 80+, respectively, p<0.001) as was the use of potentially interacting drugs (30.0%, 42.2%, 47.7%, respectively, p < 0.001). Most older adults with nocturia have medical conditions and concomitant medication use that makes very difficult the routine and safe use of the only FDA approved medications for nocturia. Providers prescribing desmopressin analogues in older adults need to carefully screen patients, employ frequent monitoring, and use shared decision-making to prevent undue harm.

